# A novel visual dynamic nomogram to online predict the risk of unfavorable outcome in elderly aSAH patients after endovascular coiling: A retrospective study

**DOI:** 10.3389/fnins.2022.1037895

**Published:** 2023-01-10

**Authors:** Wei Lu, YuLan Tong, Cheng Zhang, Lan Xiang, Liang Xiang, Chen Chen, LeHeng Guo, YaJie Shan, XueMei Li, Zheng Zhao, XiDing Pan, ZhiHong Zhao, JianJun Zou

**Affiliations:** ^1^School of Basic Medicine and Clinical Pharmacy, China Pharmaceutical University, Nanjing, China; ^2^Department of Pharmacy, Nanjing First Hospital, Nanjing Medical University, Nanjing, China; ^3^Department of Neurology, The First Affiliated Hospital (People’s Hospital of Hunan Province), Hunan Normal University, Changsha, China; ^4^Department of Pharmacy, Nanjing First Hospital, China Pharmaceutical University, Nanjing, China

**Keywords:** nomogram, aneurysmal subarachnoid hemorrhage, elderly patients, unfavorable outcome, predict

## Abstract

**Background:**

Aneurysmal subarachnoid hemorrhage (aSAH) is a significant cause of morbidity and mortality throughout the world. Dynamic nomogram to predict the prognosis of elderly aSAH patients after endovascular coiling has not been reported. Thus, we aimed to develop a clinically useful dynamic nomogram to predict the risk of 6-month unfavorable outcome in elderly aSAH patients after endovascular coiling.

**Methods:**

We conducted a retrospective study including 209 elderly patients admitted to the People’s Hospital of Hunan Province for aSAH from January 2016 to June 2021. The main outcome measure was 6-month unfavorable outcome (mRS ≥ 3). We used multivariable logistic regression analysis and forwarded stepwise regression to select variables to generate the nomogram. We assessed the discriminative performance using the area under the curve (AUC) of receiver-operating characteristic and the risk prediction model’s calibration using the Hosmer–Lemeshow goodness-of-fit test. The decision curve analysis (DCA) and the clinical impact curve (CIC) were used to measure the clinical utility of the nomogram.

**Results:**

The cohort’s median age was 70 (interquartile range: 68–74) years and 133 (36.4%) had unfavorable outcomes. Age, using a ventilator, white blood cell count, and complicated with cerebral infarction were predictors of 6-month unfavorable outcome. The AUC of the nomogram was 0.882 and the Hosmer–Lemeshow goodness-of-fit test showed good calibration of the nomogram (*p* = 0.3717). Besides, the excellent clinical utility and applicability of the nomogram had been indicated by DCA and CIC. The eventual value of unfavorable outcome risk could be calculated through the dynamic nomogram.

**Conclusion:**

This study is the first visual dynamic online nomogram that accurately predicts the risk of 6-month unfavorable outcome in elderly aSAH patients after endovascular coiling. Clinicians can effectively improve interventions by taking targeted interventions based on the scores of different items on the nomogram for each variable.

## Introduction

Aneurysmal subarachnoid hemorrhage (aSAH) is a significant cause of morbidity and mortality throughout the world (Connolly et al., 2012). Studies have shown that endovascular coiling, as a major tool for managing aSAH (Connolly et al., 2012), is associated with a better outcome compared with neurosurgical clipping (Molyneux et al., 2005, 2015; Lindgren et al., 2018; Belavadi et al., 2021). However, endovascular coiling still carries an approximately 18–48.8% risk of unfavorable outcome (Molyneux et al., 2005, 2015; Duan et al., 2016; Lindgren et al., 2018; Wang et al., 2021). And increasing age was associated with increased risk of death and unfavorable outcome (Inagawa et al., 1988; Ohkuma et al., 2017; Øie et al., 2020). Therefore, early and reliable prediction of the elderly aSAH patients’ unfavorable outcome after endovascular coiling is important in clinical practice for decision-making about treatment options but also for providing information for the patients and their families. Through prediction, clinicians can increase the opportunity for precaution, as additional attention and available resources can be distributed to such patients. To achieve this goal, risk prediction models are necessary.

Unfortunately, a dynamic nomogram model to predict the clinical prognosis of elderly aSAH patients after endovascular coiling has not been reported. In the past, Duan et al. (2016) developed an aSAH prognostic score including age, hypertension, Hunt-Hess scale, Fisher scale, aneurysm location, and periprocedural complications; The area under the receiver operating characteristic curve (AUROC) of the prognostic score was 0.864. However, this score for individualized prediction of outcome is limited by dichotomization/categorization using continuous variables such as age. The disadvantage of dichotomization is that it does not utilize within-category information, resulting in the loss of information. In addition, according to Oh et al. (2018) the including factors Hunt-Hess scale and Fisher scale, have many limitations which could lead to the initial misdiagnosis. Oh et al. (2018) also confirmed this misdiagnosis of aSAH did affect clinical outcomes negatively.

In our investigation for prediction nomograms of aSAH outcome, four prediction models were developed in whole aSAH patients, but not in elderly aSAH patients. The performance of these prediction nomograms yielded AUCs ranging from 0.784 to 0.885 (Zhang et al., 2020; Hu et al., 2021; Wang et al., 2021; Yan et al., 2021). These previous studies, although not predicting elderly patients, have proven the efficacy and potential of nomogram approaches in aSAH medical fields. Nomogram is a visualized statistical tool (Jalali et al., 2019), incorporating different data to develop a continuous scoring system that embodies the personal and accurate risk probability. Such advantage of nomogram emphasized its potential as an important component of modern medical decision-making, which has been applied to a wide range of clinical practices including cancer and acute kidney injury (Cui et al., 2020; Chen et al., 2021; Li et al., 2022). But to our knowledge, elderly aSAH patients have not been studied with nomograms to predict the risk of unfavorable outcome. To allow a reader to interact with the nomogram in a user-friendly manner, dynamic nomograms are urgently needed. Hence, accurate risk prediction dynamic nomograms in elderly aSAH patients are necessary.

Hence, we aimed to develop a simple, accurate, and clinically useful dynamic nomogram to predict the risk of 6-month unfavorable outcome in elderly aSAH patients after endovascular coiling.

## Materials and methods

### Study population

In this retrospective study, we collected elderly patients admitted to the People’s Hospital of Hunan Province for aSAH from January 2016 to June 2021. The eligibility criteria in this study were: (1) age ≥ 65 years; (2) patients diagnosed with aSAH who received endovascular coil embolization. Exclusion criteria included the followings: (1) more than 3 days from ictus to admission; (2) with blood blister-like, fusiform, or dissecting aneurysms; (3) with severe diseases in other systems which seriously affect the prognosis of disease and survival time, such as carcinoma of the pancreas, chronic or acute renal failure, liver cirrhosis, and so on. For further analysis, we excluded variables with more than 20% missing values and patients with missing features. The study was approved by the Ethics Committee of Hunan Provincial People’s Hospital [document number: (2015)-10].

### Demographic and clinical factors

We reviewed the medical and diagnostic imaging records of the elderly patients on admissions, such as demographics and clinical characteristics, attack time, aneurysmal characteristics, clinical scores used to evaluate the severity of the aSAH, and medical history, including hypertension, diabetes mellitus, coronary heart disease, and cerebral hemorrhage. In-hospital treatment and clinical assessment were also included, such as operation time, the use of drugs during operation, complications after an aneurysm ruptured (like hydrocephalus, cerebral infarction, symptomatic vasospasm), ventilated breathing status, and pupillary response. The complicated with cerebral infarction defined as patients with post-operative focal neurological deficit syndrome (such as hemiplegia, aphasia, apraxia, hemianopia, or neglect), requiring CT or MRI examination 24 h later to confirm the presence of post-operative responsible infarction corresponding to new focal neurological deficit symptoms. The functional outcome was determined according to the modified Rankin Scale (mRS) score. Acquisition of 6-month mRS was made by telephone interview and cerebral angiography at re-admission check. The data mentioned above were collected by clinicians who are well versed in the aSAH patient journey to reduce the admission of incorrect data.

### Patient outcome

According to mRS, the main outcomes of elderly patients at 6-month were divided into the favorable outcome group (mRS < 3) and the unfavorable outcome (mRS ≥ 3) group.

### Statistical analysis

Firstly, using the Shapiro–Wilk test to confirm whether the continuous data was by normal distribution or not. According to outcomes of the Shapiro–Wilk test, continuous variables were described as means with standard deviation or median value with an interquartile range. Categorical variables were reported as the number of events and the percentage of events occurring in total. Differences in continuous variables between the two groups were assessed using the *t*-test for normally distributed data or Mann–Whitney *U*-test if data was not normally distributed. As for categorical variables, we used the χ^2^-test or Fisher’s exact test to compare the differences between the two groups. The probability value <0.05 was considered statistically significant in all analyses.

### Variable selection and construction of nomogram

A nomogram model was generated to predict the risk of 6-month unfavorable outcome. All significant variables and traditionally thought to be risk factors of the unfavorable outcome were entered into a logistic regression model to carry a further selection and then construct the nomogram. Moreover, a forward stepwise regression was carried out to confirm the final variables composing the nomogram model. To enhance the predictive performance, we estimated the multicollinearity of the selected variables using the variance inflation factor (VIF), and VIF < 5 was considered non-significant. Finally, we also calculated the odds ratios (ORs) with 95% confidence intervals (95% CIs) of each variable found to be significantly associated with unfavorable outcome. By giving each of the independent predictors a preliminary visual score with a point range of 0–100, we showed the predictive model as a nomogram. For the traditional nomogram, scores of each variable can be acquired by drawing a line from the position on the relevant axis to the points axis. The points allotted to each predictor are then summed to determine the final point. Finally, we can acquire the risk of 6-month unfavorable outcome by converting the total point. To make this process more convenient and easier, we also constructed the dynamic nomogram.

### Validation of nomogram

We use discrimination, calibration, and clinical usefulness to evaluate the performance of the established prediction model. Discrimination of the model describes the ability to separate patients with unfavorable versus favorable outcome, and calibration shows the ability to predict the actual outcome in patients. The AUROC with a corresponding 95% CI, which is a common measure, was used to quantify discrimination of the nomogram model, and we used Hosmer–Lemeshow test to assess calibration. When the model’s predictive value matches the patient’s actual risk, a 45° line indicates perfect calibration. To prove the clinical usefulness, constructions of the decision curve analysis (DCA) and the clinical impact curve (CIC) are needed. In order to prevent the overfitting bias, we also conducted an internally validated for the nomogram through the bootstrap validation method (*n* = 1000). What’s more, the characteristics of the nomogram such as specificity, sensitivity, and so on were calculated.

The statistical analysis and model visualization was conducted using the R software (version 4.1.3, R Development Core Team, Auckland, New Zealand)^1^ and SPSS version 25.0 (IBM Corporation, Armonk, NY, USA) statistical software.

## Results

### Clinical characteristics

In this study, 213 patients with aSAH enrolled in our study. After excluding four patients with missing variables, the final dataset included 209 elderly patients. The cohort’s median age was 70 (interquartile range: 68–74) years, and 27.8% of them (58) were men. According to the clinical outcome, favorable outcome was observed in 133 patients (63.6%, median age: 70 years, number of male patients: 37), and in the unfavorable outcome group, there had 76 patients (36.4%, median age: 72 years, number of male patients: 21).

### Variable selection

Table 1 shows the clinical, demographic, treatment-related information, and laboratory characteristics of patients in the favorable and unfavorable outcome groups. Age, systolic blood pressure (SBP), diastolic blood pressure (DBP), creatinine, D-dimer, fasting blood glucose (FBG), blood urea nitrogen (BUN), white blood cell count (WBC), aspartate transaminase (AST), neutrophil-to-lymphocyte ratio (NLR, NEU/LYMPH), lymphocyte-to-monocyte ratio (LMR, LYMPH/MON), systemic inflammation response index (SIRI, NEU × MON/LYMPH), systemic immune-inflammation (SII, PLT × NEU/LYMPH), fibrinogen degradation product (FDP), lactate dehydrogenase (LDH), modified Fisher score, Hunt and Hess score, located in the artery bifurcation, multiple aneurysms, hydrocephalus, external ventricular drainage (EVD), complicated with cerebral infarction, symptomatic cerebral vasospasm, complicated with pulmonary infection, using a ventilator, ventricular hematocele, pupillary reflex, complicated with cerebral hemorrhage, rebleeding were statistically significant variables in the univariate analysis.

**TABLE 1:** Clinical, demographic, and laboratory data of patients with aSAH treated by endovascular coiling.

Variables	Favorable outcome (*n* = 133, 63.6%)	Unfavorable outcome (*n* = 76, 36.4%)	*P*-values
Male, sex, *n* (%)	37 (27.8%)	21 (27.6%)	1.000
Age, years, median (IQR)	70 (67–72)	72 (69–76)	0.001*^†^
Medical history, *n* (%)			
Hypertension	95 (71.4%)	56 (73.7%)	0.849
Diabetes	16 (12.0%)	14 (18.4%)	0.288
Hyperlipidemia	42 (31.6%)	25 (32.9%)	0.966
Coronary heart disease	29 (21.8%)	20 (26.3%)	0.568
Atrial fibrillation	3 (2.3%)	3 (3.9%)	0.784
Cerebral ischemia history	4 (3.0%)	7 (9.2%)	0.107
Cerebral hemorrhage history	2 (1.5%)	2 (2.6%)	0.962
Aneurysms rupturing many times	1 (0.8%)	1 (1.3%)	1.000
Smoking, *n* (%)	21 (15.8%)	11 (14.5%)	0.957
Alcohol, *n* (%)	11 (8.3%)	5 (6.6%)	0.863
Baseline data, median (IQR)			
SBP, mmHg	144 (132–162)	155 (135–172)	0.033*^†^
DBP, mmHg	80 (70–86)	84 (74–96)	0.042*^†^
FBG, mmol/L	6.80 (5.80–7.99)	7.92 (6.83–9.89)	<0.001*^†^
Homocysteine, μmol/L	10.84 (9.01–13.75)	11.60 (9.25–16.88)	0.121
Total cholesterol, mmol/L	4.55 (4.02–5.28)	4.68 (4.00–5.39)	0.428
Triglyceride, mmol/L	1.45 (0.97–2.18)	1.54 (1.08–2.15)	0.560
LDL, mmol/L	2.64 (2.15–3.11)	2.74 (2.18–3.16)	0.375
Creatinine, μmol/L	54.38 (45.67–67.00)	59.84 (49.98–80.98)	0.045*^†^
Uric acid, μmol/L	259.30 (190.40–328.00)	276.75 (207.52–350.75)	0.189
BUN, mmol/L	4.65 (3.58–5.43)	5.38 (3.84–6.96)	0.003*^†^
WBC, × 10^9/L	9.60 (7.74–12.88)	12.66 (10.16–15.83)	<0.001*^†^
NLR	9.66 (5.88–16.23)	13.34 (8.96–19.81)	0.003*^†^
LMR	2.31 (1.55–3.31)	1.79 (1.16–2.37)	0.001*^†^
PLR	205.69 (164.08–304.29)	217.20 (146.30–329.64)	0.770
SIRI,/L	3.59 (1.97–6.50)	6.52 (3.74–11.16)	<0.001*^†^
SII,/L	1914.23 (1171.31–3310.49)	2475.23 (1502.54–3581.17)	0.018*^†^
Hemoglobin, g/L	125.00 (113.00–133.00)	120.00 (112.75–131.25)	0.337
Prealbumin, g//L	218.00 (193.00–256.00)	228.40 (184.15–263.25)	0.767
ALT, U/L	17.00 (11.90–22.10)	16.95 (13.62–24.92)	0.208
AST, U/L	21.29 (17.05–28.01)	27.97 (20.16–35.16)	0.001*^†^
TBIL, μmol/L	14.30 (11.30–17.63)	14.90 (11.33–18.55)	0.415
DBIL, μmol/L	4.40 (3.30–5.50)	4.71 (3.38–5.85)	0.275
IDBIL, μmol/L	9.84 (7.80–12.40)	9.80 (7.90–12.98)	0.645
ALP, U/L	71.10 (59.00–88.00)	72.25 (62.00–87.32)	0.689
INR	0.90 (0.86–0.95)	0.91 (0.86–0.98)	0.339
D-dimer, μg/ml	1.69 (1.04–3.12)	3.62 (1.68–7.77)	<0.001*^†^
FDP, mg/L	6.20 (3.80–11.00)	12.05 (6.57–21.15)	<0.001*^†^
PT, s	10.20 (9.80–10.80)	10.40 (9.90–11.20)	0.172
APTT, s	24.40 (22.50–26.70)	23.95 (22.08–26.58)	0.563
Thrombin time, s	17.80 (16.70–18.70)	18.00 (16.88–19.62)	0.164
AFR	14.03 (11.69–16.43)	14.41 (11.42–16.73)	0.831
LDH, U/L	199.40 (172.58–237.77)	227.57 (193.92–266.33)	0.001*^†^
Characteristics of aneurysm			
Size of aneurysm, *n* (%)			0.124^†^
Max diameter <5 mm	57 (42.9%)	28 (36.8%)	
Max diameter 5–10 mm	69 (51.9%)	38 (50.0%)	
Max diameter 10–25 mm	7 (5.3%)	10 (13.2%)	
Max diameter >25 mm	0 (0.0%)	0 (0.0%)	
Shape of Aneurysm, *n* (%)			0.769
Quasi-circular	50 (37.6%)	26 (34.2%)	
Irregular	64 (48.1%)	42 (55.3%)	
Long-strip	8 (6.0%)	2 (2.6%)	
Lobulated	4 (3.0%)	2 (2.6%)	
Ellipse	7 (5.3%)	4 (5.3%)	
Location of aneurysm, *n* (%)			0.090^†^
Anterior circulation	127 (95.5%)	67 (88.2%)	
Posterior circulation	6 (4.5%)	9 (11.8%)	
Arterial bifurcation, *n* (%)	9 (6.8%)	13 (17.1%)	0.035*^†^
Wide neck, *n* (%)	96 (72.2%)	61 (80.3%)	0.257
Multiple aneurysms, *n* (%)	25 (18.8%)	30 (39.5%)	0.002*^†^
Hunt-Hess grade on admission, *n* (%)			<0.001*^†^
0	2 (1.5%)	0 (0.0%)	
I	4 (3.0%)	2 (2.6%)	
II	88 (66.2%)	25 (32.9%)	
III	30 (22.6%)	22 (28.9%)	
IV	9 (6.8%)	26 (34.2%)	
V	0 (0.0%)	1 (1.3%)	
WFNS grade on admission			0.379
I	77 (57.9%)	44 (57.9%)	
II	14 (10.5%)	6 (7.9%)	
III	5 (3.8%)	2 (2.6%)	
IV	27 (20.3%)	12 (15.8%)	
V	10 (7.5%)	12 (15.8%)	
Modified Fisher score, median (IQR)	2 (2–2)	4 (2–4)	<0.001*^†^
Pre-operative using anticoagulant drugs, *n* (%)	16 (12.0%)	9 (11.8%)	1.000
Intraoperative using tirofiban, *n* (%)	3 (2.3%)	6 (7.9%)	0.115
Time from aneurysm ruptured to surgery, h, median (IQR)	47.00 (26.00–74.47)	37.50 (18.83–60.69)	0.084
Embolization mode, *n* (%)			0.778
Simple embolization	53 (39.8%)	28 (36.8%)	
Stent assistance	80 (60.2%)	48 (63.2%)	
Complication, *n* (%)			
Hydrocephalus	9 (6.8%)	17 (22.4%)	0.002*^†^
EVD	6 (4.5%)	22 (28.9%)	<0.001*^†^
Cerebral infarction	7 (5.3%)	22 (28.9%)	<0.001*^†^
Cerebral vasospasm	16 (12.0%)	28 (36.8%)	<0.001*^†^
Intracranial infection	1 (0.8%)	4 (5.3%)	0.114
Pulmonary infection	65 (48.9%)	71 (93.4%)	<0.001*^†^
Cerebral hernia	0 (0.0%)	13 (17.1%)	<0.001*^†^
Deep venous thrombosis	7 (5.3%)	9 (11.8%)	0.147
Ventricular hemorrhage	52 (39.1%)	55 (72.4%)	<0.001*^†^
Cerebral hemorrhage	5 (3.8%)	10 (13.2%)	0.024*^†^
Rebleeding	4 (3.0%)	16 (21.1%)	<0.001*^†^
Using a ventilator, *n* (%)	3 (2.3%)	40 (52.6%)	<0.001*^†^
Pupillary reflex, *n* (%)			<0.001*^†^
Sensitive	118 (88.7%)	27 (35.5%)	
Dull	15 (11.3%)	20 (26.3%)	
Disappear	0 (0.0%)	29 (38.2%)	

aSAH, aneurysmal subarachnoid hemorrhage; SBP, systolic blood pressure; DBP, diastolic blood pressure; FBG, fasting blood glucose; LDL, low density lipoprotein; BUN, blood urea nitrogen; WBC, white blood cell; NLR, neutrophil-to-lymphocyte ratio; LMR, lymphocyte-to-monocyte ratio; PLR, platelet-to-lymphocyte ratio; SIRI, systemic inflammation response index; SII, systemic immune-inflammation; ALT, alanine transaminase; AST, aspartate aminotransferase; TBIL, total bilirubin; DBIL, direct bilirubin; IDBIL, indirect bilirubin; ALP, alkaline phosphatase; INR, international normalized ratio; FDP, fibrinogen degradation product; PT, prothrombin time; APTT, activated partial thromboplastin time; AFR, albumin-fibrinogen ratio; LDH, lactate dehydrogenase; WFNS, World Federation of Neurosurgical Societies; EVD, external ventricular drainage; IQR, interquartile range.

*P < 0.05; ^†^indicates the variable entered into the logistic regression model.

SIRI and SII index are defined as follows: SIRI = neutrophil count × monocyte count/lymphocyte count, SII = platelet count × neutrophil count/lymphocyte count.

The concept of using a ventilator excludes the use in interventional surgery.

**FIGURE 1:**
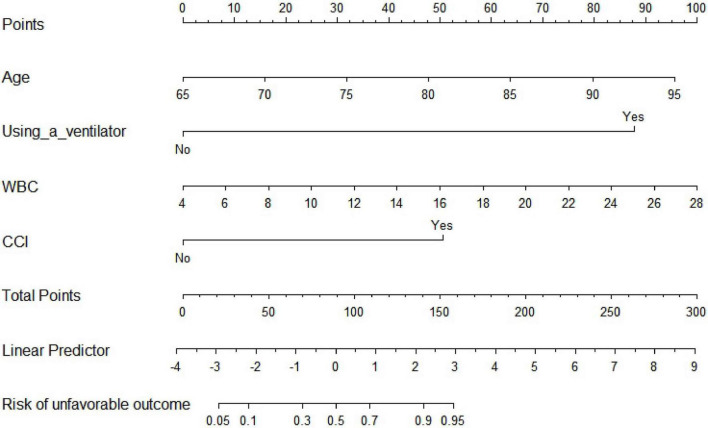
Traditional nomogram for predicting 6-month unfavorable outcome in elderly cohort with aneurysmal subarachnoid hemorrhage (aSAH) treated by endovascular coiling. WBC, white blood cell count; CCI, complicated with cerebral infarction.

Thirty-one variables (including traditional aSAH prognostic risk factors such as size and position of the aneurysm) entered the multivariate analysis. As shown in Table 2, after multivariate analysis and forward stepwise regression analysis, age (OR: 1.136, 95% CI: 1.048–1.237, *p* = 0.002), using a ventilator (OR: 36.392, 95% CI: 10.894–170.712, *p* < 0.001), WBC (OR: 1.190, 95% CI: 1.062–1.343, *p* = 0.003), complicated with cerebral infarction (OR: 5.885, 95% CI: 1.767–20.849, *p* = 0.004) were still the independent risk predictors of 6-month unfavorable outcome and finally incorporated into a logistic regression model to establish the nomogram that predicted the risk of 6-month unfavorable outcome in elderly patients with aSAH who received endovascular coil embolization. The nomogram is shown in Figure 1. None of the variables we chose showed significant statistical collinearity.

**TABLE 2:** Significant predictors of 6-month unfavorable outcome of patients with aSAH treated by endovascular coiling.

	OR	Error	Wald	*P-*value	95% CI
Age	1.136	0.042	9.143	0.002	1.048–1.237
Using a ventilator	36.392	0.683	27.689	0.000	10.894–170.712
WBC	1.190	0.060	8.495	0.003	1.062–1.343
CCI	5.885	0.624	8.064	0.004	1.767–20.849

aSAH, aneurysmal subarachnoid hemorrhage; WBC, white blood cell; CCI, complicated with cerebral infarction.

### Construction of nomogram

**FIGURE 2:**
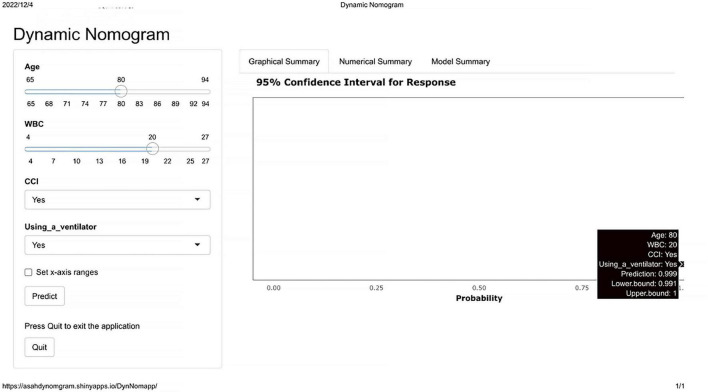
Dynamic nomogram for predicting 6-month unfavorable outcome in elderly cohort with aneurysmal subarachnoid hemorrhage (aSAH) treated by endovascular coiling. WBC, white blood cell count; CCI, complicated with cerebral infarction.

As shown in Figure 1, the nomogram assigns >95% risk of unfavorable outcome in an aSAH patient aged 80 years (48 points) after endovascular coiling who is using a ventilator (88 points), with a WBC count of 20 × 10^9 (66 points) and cerebral infarction (50 points), then the total score is 252 points. On the contrary, <10% risk of unfavorable outcome is indicated for a patient who is 67 years old (10 points), does not use a ventilator (0 points), with a WBC count of 6 × 10^9 (8 points), and without cerebral infarction (0 points), then the total score is 18 points. The nomogram can then more precisely reclassify the risk of unfavorable outcome by transforming the total points into a continuum of individual probability. And the dynamic nomogram page is shown in Figure 2. For prognosis, a free online calculator and nomograms are accessible for all clinicians and patients at https://asahdynomgram.shinyapps.io/DynNomapp/.

### Validation of nomogram

**FIGURE 3:**
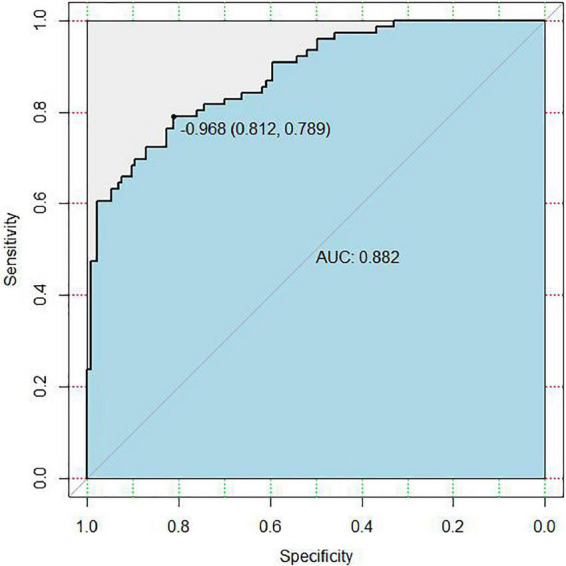
Receiver operating characteristic (ROC) curve of the nomogram for predicting 6-month unfavorable outcome in elderly cohort with aneurysmal subarachnoid hemorrhage (aSAH) who received endovascular coil embolization. AUC, the area under the curve.

**FIGURE 4:**
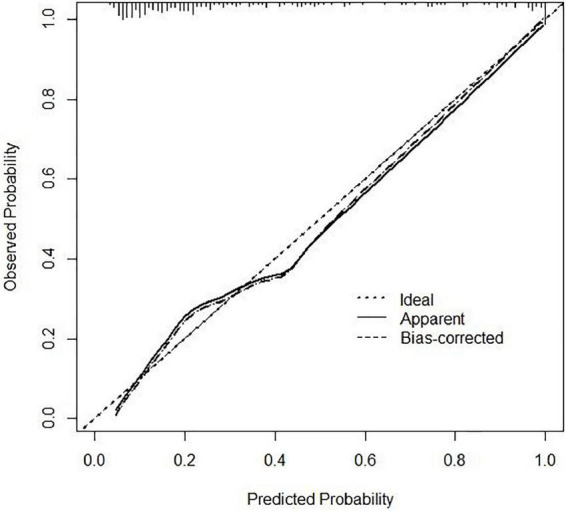
Calibration curve of the nomogram. The *x*-axis represents the nomogram–predicted risk and the *y*-axis represents the actual risk of 6-month unfavorable outcome. The 45° dashed line is a reference line that shows perfect prediction. The dotted line is the performance of the nomogram, while the solid line corrects for any bias in the nomogram.

**FIGURE 5:**
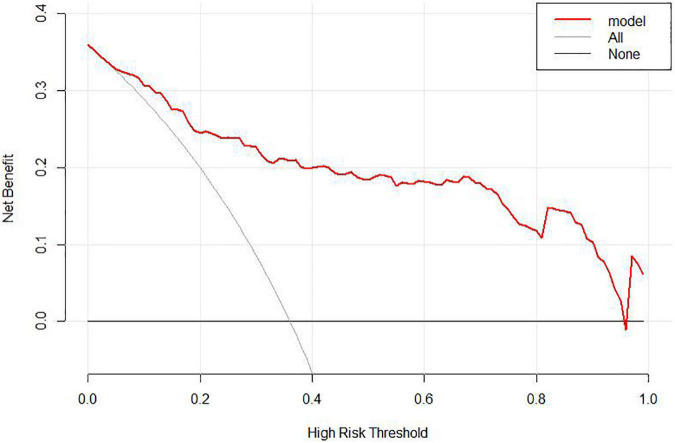
Decision curve analysis (DCA) of the nomogram. When the risk of unfavorable outcome generated by the nomogram was between 0.05 and 0.95, DCA reveals that the nomogram confers more benefit than either the treat-all or treat-none strategy.

**FIGURE 6:**
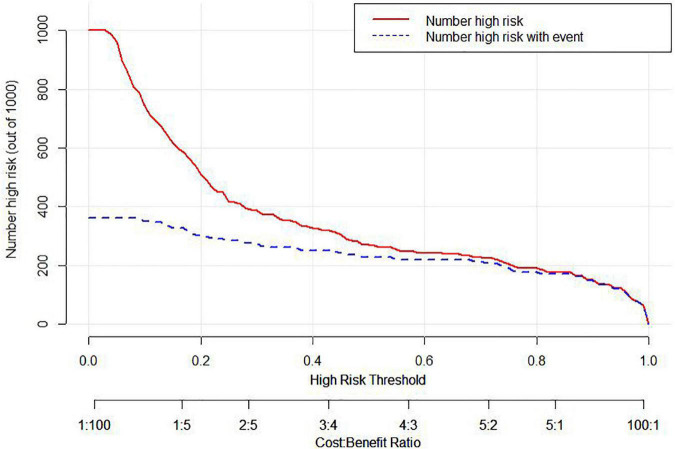
Clinical impact curve (CIC) of the nomogram. The Number high risk curve indicates the number of patients classified as high risk by the nomogram under each threshold probability. The Number high risk with event curve stands for actual numbers under each threshold probability.

In the area under the curve (AUC), the receiver operator characteristic (ROC) of the nomogram was 0.882 (95% CI: 0.835–0.929) in the elderly patients (Figure 3), which showed good discrimination of the nomogram. The characteristics of the predictive model can be found in Table 3. Moreover, the calibration curve demonstrated that the result predicted by the nomogram was consistent with the observed result (Figure 4). We could know that the calibration of the nomogram (*p* = 0.3717) was good according to the Hosmer–Lemeshow goodness-of-fit test. The results of DCA showed that the use of the nomogram to predict 6-month unfavorable prognosis when the threshold probability was between 5 and 95% added more net benefit than the “treat all” or “treat none” strategies (Figure 5). In this example, if a risk threshold of 20% were established, patients consented to some treatment measures if the risk of 6-month unfavorable outcome was more than 20% and would gain about 0.25 net benefits. The CIC displayed real statistics for each risk threshold along with the expected number of high-risk individuals predicted by the nomogram (Figure 6). If we screened 1,000 patients and set a 20% risk threshold, for instance, 510 patients would be expected to be high risk, but only roughly 300 patients really met this criterion. In addition, according to the result of the bootstrap validation method, the nomogram was internally validated with an AUROC of 0.876 (95% CI: 0.829–0.923) and proved the accuracy of predictive ability.

**TABLE 3:** The characteristics of the predictive model.

	AUROC (95% CI)	Specificity	Sensitivity	PPV	NPV	Accuracy
Model	0.835–0.929	81.2%	78.9%	70.6%	87.1%	80.4%

AUROC, the area under the receiver operating characteristic curve; PPV, positive predictive value; NPV, negative predictive value.

## Discussion

This study is the first dynamic nomogram that accurately predicts the risk of 6-month unfavorable outcome in elderly aSAH patients after endovascular coiling. To compare our nomogram discriminative performance (AUC = 0.882) with other nomograms targeting the same unfavorable outcome, we investigated published nomogram studies in whole aSAH patients (nomograms in elderly aSAH patients have not been studied). Our nomogram showed better performance. To our knowledge, four nomograms were developed and the performance of the nomograms yielded AUCs ranging from 0.784 to 0.885 (Zhang et al., 2020; Hu et al., 2021; Wang et al., 2021; Yan et al., 2021). Two nomograms reached an AUC of 0.831 and 0.851 for incorporating free fatty acids and SIRI, respectively (Zhang et al., 2020; Wang et al., 2021). Apart from these, reached an AUC of 0.784 for a traditional nomogram with 9 variables (Yan et al., 2021). Our nomogram showed better performance, in spite of requiring only 4 variables for calculating probability versus 5 and 9. Our nomogram might seem consistent with the published performance of the dynamic nomogram (AUC 0.885) (Hu et al., 2021). Smaller data, however, was used for establishing the dynamic nomogram compared with our nomogram. To the best of our knowledge, this model is the first to reach objectively measured AUC of 0.882 in elderly aSAH patients undergoing endovascular coiling surgery. Furthermore, combining our nomogram and clinicians reliably further improves model performance.

**FIGURE 7:**
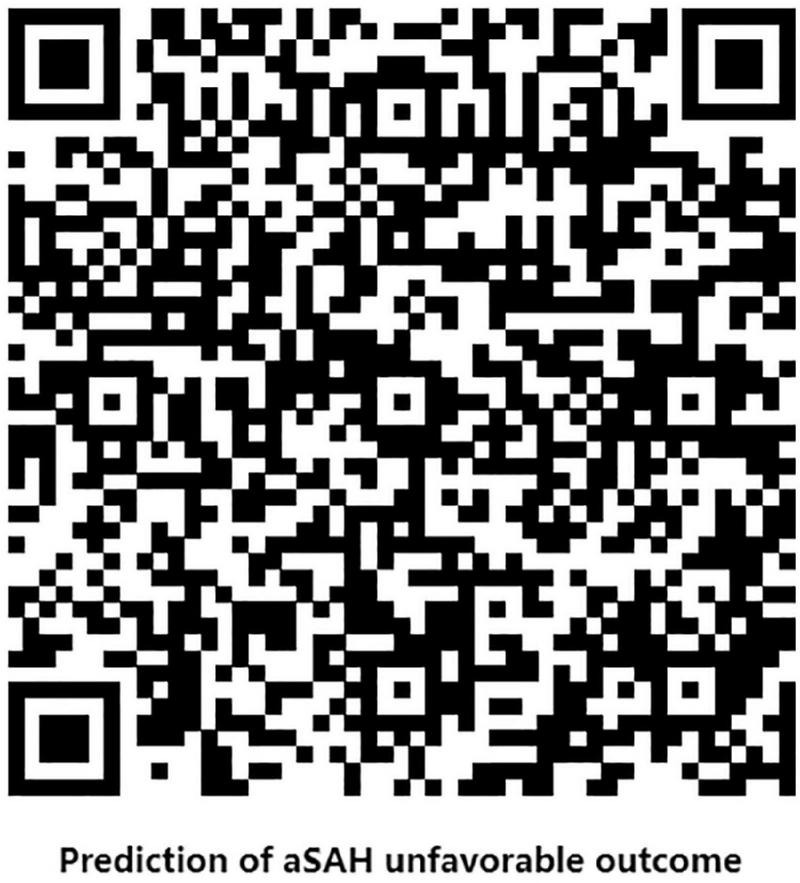
The QR code of the nomogram for predicting 6-month unfavorable outcome in elderly cohort with aneurysmal subarachnoid hemorrhage (aSAH) who received endovascular coil embolization.

A strength of our study is that automatically predicting the probability of the unfavorable outcome. Such superiorities make the dynamic nomogram a more accurate and practical tool compared to traditional predictive models. Physicians could take targeted interventions on the basis of the score of different items on the nomogram for each variable, improving the interventions efficiently. However, non-professionals may think traditional nomogram unsuitable because it requires manual calculation. Given this situation, we established an online dynamic nomogram.^2^ In order to further facilitate promotion and use we also provide the quick response code (QR) code (Figure 7). The eventual accurate value of unfavorable outcome risk with 95% CI could be automatically calculated immediately according to the input value of each variable in the dynamic nomogram. Clinicians could visit the website directly on their cell phones or computer whenever and wherever. This makes the dynamic nomogram easy to apply for clinical assistance and it is a promising tool for clinical prognostication. Risk prediction could enable patient risk stratification, entitling clinicians to focus on high-risk patients. The application of the nomogram offers the possibility of personalized identification for the elderly.

Another strength of this study is that DCA and CIC were used to evaluate the clinical utility of our nomogram. Clinicians hold an intuition from years of treating patients *via* which they have developed their qualifications (although subjective) to forecast results. Even experienced clinicians are limited by the inability to handle multiple variables at the same time. But based on DCA and CIC metrics clinicians can set a risk threshold; that is, to determine who should accept some treatment intervention to prevent the development of 6-month unfavorable outcome, and how can minimize the unreasonable waste of medical resources in clinical practice. The nomogram demonstrated by DCA in our study, the DCA and CIC, had good clinical utility. The higher risk of developing unfavorable outcome, the more stringent interventions should be taken. Therefore, our study could help decrease the incidence of unfavorable outcome in high-risk aged patients through targeted interventions.

The third strength of this study is that the factors included in our dynamic nomogram are easily obtained in clinical management. What is more, all these variables do not like aneurysm size and Fisher grade which are sensitive to error in measurement (van Donkelaar et al., 2019). Unfavorable outcome occurs frequently among elderly aSAH patients (Inagawa et al., 1988; Ohkuma et al., 2017; Øie et al., 2020), and preventive measures should be taken as soon as possible after the onset of aSAH. Given this situation, variables included in the nomogram should be reliable and available. Consistent with previous research, our study showed that the higher age level (Jaja et al., 2018; van Donkelaar et al., 2019; de Jong et al., 2021; Yan et al., 2021) and complicated with cerebral infarction (Rosengart et al., 2007) were independent predictor for the unfavorable 6-month outcome of elderly aSAH patients following endovascular coiling. Increasing patient age by steps of 10 years was correlated with low Glasgow Coma Score (GCS) (Zumofen et al., 2018). Patient age, independently predicted worse admission scores, which in turn foretells poor outcome following aSAH (Rosen and Macdonald, 2005; Jaja et al., 2013). Zumofen et al. (2018) found in their cohort that the older was a patient, the lower was his pre-ictal functional status, and the more likely he presented in poor condition following aSAH. In recent decades, the systemic inflammatory response has attracted the clinician’s attention for assessing aSAH severity and predicting the prognosis (Kalinkin et al., 2017; Gusdon et al., 2021; Ma et al., 2021); our study confirmed that the risk of developing unfavorable outcome is greater in patients with higher WBC at the time of admission. Compared with other research which revealed the prognostic value of the NLR and SIRI in patients with aneurismal subarachnoid hemorrhage (Kalinkin et al., 2017; Zhang et al., 2020; Hu et al., 2021), the WBC on admission is more intuitive and accessible. Finally, using a ventilator after endovascular coiling is included in the nomogram for elderly patients with aSAH. The relationship between breathing status and unfavorable outcomes of aSAH patients was also reported in previous studies (Zheng et al., 2019; Xia et al., 2020). That is probably owing to a bad physical condition, and more disease severity was prone to have a poor outcome (Zheng et al., 2019). In comparison, ventilated breathing status is related to physical condition and severity of illness of aSAH patients. Zafar et al. (2018) also reported that pulmonary complications of aSAH predictors included duration of mechanical ventilation. For example, in patients without lung injury, who require ventilation for depressed arousal, over ventilation can result in hyperoxia or hypocapnea. And exposure to excess oxygen after SAH may represent a modifiable factor for morbidity and mortality in this population (Jeon et al., 2014). In addition, hypocapnia is independently associated with poor functional outcomes and symptomatic vasospasm (Solaiman and Singh, 2013). Thus, our findings further highlighting the untapped value of using a protocolized approach to ventilator management in aSAH patients. In spite of some scales, such as the World Federation of Neurosurgical Societies (WFNS) grade, Hunt-Hess grade, and Modified Fisher grade, were not included in our model, the performance of our nomogram model did not degrade due to not include the three variables mentioned above and the superior data handling capabilities of the nomogram model. In this regard, the following explanations are given. (i) Difficulty assessing verbal responses in such patients if the patient is intubated (Mack et al., 2008). (ii) Intubation, sedation might obscure the clinical status of patients with poor-grade SAH and the quality of assessments, or negatively influence the predictive value of the WFNS grade (Fung et al., 2016). (iii) The Hunter-Hess and Fisher scales have many limitations that may lead to initial misdiagnosis (Oh et al., 2018). WFNS grade, Hunt-Hess grade, and Modified Fisher grade were not included in this nomogram, making it an objective and simple-to-use tool to evaluate patients for unfavorable outcome in a dynamic online manner responding to a drive toward a more personalized medicine with a user-friendly digital interface that may help contributing to a better clinical decision making. In general, based on this tool, clinicians can precisely treat and manage elderly aSAH patients, including neuro-intensive care, anti-inflammatory drugs, rehabilitation treatment, and treatment of cerebral infarction, and help them make clinical decisions. In other words, the clinical application of nomograms can prepare clinicians to triage high-risk elderly patients with unfavorable outcome to an appropriate level of clinical care.

However, our research also has some limitations. First, it was a single-center retrospective study and not an RCT designed to investigate the crucial critical factors influencing unfavorable 6-month outcomes. Secondly, no external validation was performed in our study; the universality performance of the nomogram deserves investigation in patients treated at other institutions. Future investigations can explore whether this model can be generalized to other institutions. Finally, the number of 209 patients in our study was small, a prospective study with a larger number of cases is needed to validate our present findings. Despite these limitations, as far as we know, our study is the first dynamic nomogram to predict the risk of unfavorable outcome in elderly patients with aSAH after endovascular coiling.

## Conclusion

This study is the first visual dynamic online nomogram with favorable discrimination ability to predict the risk of 6-month unfavorable outcome in elderly aSAH patients after endovascular coiling. Clinicians can effectively improve interventions by taking targeted interventions based on the scores of different items on the nomogram for each variable. It has more practical value for primary hospitals or inexperienced doctors. We hope our nomogram will benefit precise treatment and patient management and help clinicians make clinical decisions.

## Data availability statement

The original contributions presented in this study are included in the article/Supplementary material, further inquiries can be directed to the corresponding authors.

## Ethics statement

The studies involving human participants were reviewed and approved by the Ethics Committee of Hunan Provincial People’s Hospital [document number: (2015)-10]. The patients/participants provided their written informed consent to participate in this study.

## Author contributions

ZZ and JZ: study conception and design. WL, YT, and CZ: analysis and interpretation of data. WL and YT: drafting of the manuscript. CZ, LiX, LaX, LG, YS, XL, and ZZ: data collection. ZHZ, CC, XP, and JZ: critical revision of the manuscript for important intellectual content. All authors read and approved the final manuscript.

## Footnotes

1
https://www.r-project.org/

2
https://asahdynomgram.shinyapps.io/DynNomapp/
